# Predation pressure shapes brain anatomy in the wild

**DOI:** 10.1007/s10682-017-9901-8

**Published:** 2017-05-12

**Authors:** Alexander Kotrschal, Amy E. Deacon, Anne E. Magurran, Niclas Kolm

**Affiliations:** 1 Department of Ethology/Zoology, Stockholm University, Svante Arheniusväg 18B, 10691 Stockholm, Sweden; 2 Department of Life Sciences, The University of the West Indies, St Augustine, Trinidad and Tobago; 3 School of Biology, University of St Andrews, St Andrews, Scotland, UK

**Keywords:** Brain anatomy, Brain size, Cognitive ability, Guppy, Predation

## Abstract

There is remarkable diversity in brain anatomy among vertebrates and evidence is accumulating that predatory interactions are crucially important for this diversity. To test this hypothesis, we collected female guppies (*Poecilia reticulata*) from 16 wild populations and related their brain anatomy to several aspects of predation pressure in this ecosystem, such as the biomass of the four major predators of guppies (one prawn and three fish species), and predator diversity (number of predatory fish species in each site). We found that populations from localities with higher prawn biomass had relatively larger telencephalon size as well as larger brains. Optic tectum size was positively associated with one of the fish predator’s biomass and with overall predator diversity. However, both olfactory bulb and hypothalamus size were negatively associated with the biomass of another of the fish predators. Hence, while fish predator occurrence is associated with variation in brain anatomy, prawn occurrence is associated with variation in brain size. Our results suggest that cognitive challenges posed by local differences in predator communities may lead to changes in prey brain anatomy in the wild.

## Introduction

Predation is a major force of natural selection. After all, most species are subject to the risk of being eaten during at least some part of their life. In response to predation, animals evolve counter measures including aposematic coloration (Mappes et al. 2005), body armour (Walls and Ketola 1989), or changes in life history (Reznick 1982). Most prominently, predator–prey interactions select for numerous behavioural adaptations (Caro 2005). For instance, increased predation risk prompts chimpanzees (*Pan troglodytes*) to build their sleeping nests higher up in trees (Pruetz et al. 2008), larval anurans to reduce their overall activity levels (Relyea 2001), and fishes to form more synchronized schools (Pitcher 1995). There is a tight association between behavioural variation and brain anatomy, as across species, brain size predicts problem-solving abilities in mammalian carnivores (Benson-Amram et al. 2016) and self-control in homeothermic vertebrates (MacLean et al. 2014), and within species, brain size is positively associated with learning ability (Kotrschal et al. 2013a, b, 2014a). Therefore, as predation selects for behavioural adaptations and those are produced by the brain, predation should be important for brain evolution (e.g. van der Bijl and Kolm 2016). Indeed, the fossil record suggests that ungulates evolved larger brains in the presence of carnivores (Jerison 1973). In fishes, a recent analysis of 623 predator–prey pairs revealed that brains of prey species were relatively larger than those of non-prey species. Moreover, in these predator–prey pairs, the size of prey and predators’ brains were correlated, suggesting a cognitive arms race (Kondoh 2010). Here, we adhere to the broad definition of ‘cognition’ as comprising all mechanisms that animals have for taking in information through the senses, retaining it, and using it to adjust behaviour to local conditions (Kotrschal and Taborsky 2010; Shettleworth 2010). Recent findings further highlight the link between predation ecology and cognition (i.e. brain size) at several levels. From the predator side, fishes at higher trophic positions in a lacustrine food web have larger brains (Edmunds et al. 2016). From the prey side, the cognitive advantage of a larger brain can lead to increased survival. This was recently shown in guppies (*Poecilia reticulata*) that were artificially selected for large and small brain size where large-brained females survived better under predation in a semi-natural setting (Kotrschal et al. 2015a). However, Walsh et al. (2016) found that in two areas where killifish (*Rivulus hartii*) co-occur with predatory fish species, males tended to develop smaller brains than in two adjacent areas where the killifish was not under threat from fish predators. It is clear that additional studies are needed to fully understand the functional associations between predation and the nervous system. Ideally, such studies should be conducted on wild populations, include detailed data on all key predator species, and use well-replicated designs.

Trinidadian guppies have become a model species for studying the interaction between ecology, especially predation pressure, and the evolution of a range of traits. This is due to the ‘natural experiment’ that exists in the mountainous regions of Trinidad’s Northern range (Haskins et al. 1961). Parallel rivers harbouring guppy populations are often interrupted by waterfalls, which prevent larger fish from venturing upstream. For small fish such as guppies, predation pressure therefore tends to be “low” above waterfalls (where large fish predators are absent) and “high” below them (where fish predators can be abundant; Haskins et al. 1961). Indeed, over the last few decades studies have revealed a great number of differences between guppies from low and high predation sites, such as coloration (Endler 1980), life history (Reznick et al. 2001), mate choice (Godin and Briggs 1996), and foraging behaviour (Fraser and Gilliam 1987). While this system has proven incredibly fruitful, it also has limitations. First, it does not facilitate investigation of more nuanced differences in predation pressure. Second, other habitat traits besides predator abundance may differ systematically between above- and below-waterfall habitats and confound the effect of predation. For example, high predation sites can be more productive (Arendt and Reznick 2005). Third, guppies are also targeted by several species of large carnivorous and omnivorous prawns (*Macrobrachium* spp.; Coat et al. 2009; Endler 1978), and these negotiate waterfalls with ease. Fish and prawn predators likely differ in their way of capturing prey and they are known to exert different selective pressures on guppy traits (Endler 1991; Millar et al. 2006).

Here we take advantage of the recent and most complete investigation of Trinidadian river biodiversity to date (Deacon et al. 2015) to investigate the effect of predation on brain anatomy in wild populations. We do this by relating data on predator community composition to brain anatomy of guppies from 16 wild populations that are closely matched in stream characteristics (Deacon et al. 2015). Because a larger brain confers a cognitive advantage and so improves predator-related performance (Kotrschal et al. 2015a; van der Bijl et al. 2015), we predict that increased predation pressure selects for larger brains. This would result in a positive association across populations between brain size and the abundance of individual predators. Predator species differ in their hunting tactics (Belgrad and Griffen 1828) and a larger brain may confer the behavioural flexibility necessary in predator-diverse habitats (Sol and Lefebvre 2000). Hence, predator species richness (i.e. predator diversity) and brain size may be positively associated. However, some aspects of cognition may be especially targeted by selection under increased predation risk and such variation in the strength of selection may result in brain regions evolving differently, i.e. in a mosaic evolution manner (Finlay et al. 2001; Kotrschal et al. 2012a; Noreikiene et al. 2015; Striedter 2005).

Even though the function of the separate brain regions is still only partly understood and single regions sometimes have multiple functions, we can make predictions about brain region sizes based on previous findings from lesion studies and neuro-ecology studies (Barton and Harvey 2000; Gonda et al. 2009; Gonzalez-Voyer et al. 2009; Kolm et al. 2009; Kotrschal et al. 1998, 2012b; Kotrschal and Palzenberger 1992; Zeng et al. 2016). For instance, the telencephalon integrates complex information and is vital in learning and memory (Striedter 2005). Learning about and remembering the location of a predator should increase a guppy individual’s survival. Predation by species that preferentially hunt from cover, such as prawns or pike cichlids may therefore select for a larger telencephalon. Also, camouflaged and crepuscular predators such as wolf fish and prawns may be spotted more accurately or earlier with a better visual system. Wolf fish and prawn predation may therefore select for larger optic tectum. Additionally, better motor skills may yield survival benefits when facing predators that actively pursue prey as the cichlids do. Guppy populations experiencing high cichlid densities may therefore have larger cerebellum (the region controlling spatial swimming skills) and/or larger medulla oblongata (where most efferent motor neurons originate). As explained above, higher predator species richness, via a greater number of cognitively challenging hunting tactics, may demand higher behavioural flexibility. We therefore predict a positive association between telencephalon size and number of predator species.

## Materials and methods

### Sampling methods

The data on predation pressure was originally collected for a study on temporal patterns of biodiversity and the impact of human recreational use of rivers on community ecology in Trinidad’s Northern Range; see Deacon et al. (2015) for detailed sampling protocol. In brief, 16 sites (50 m stretches), closely matched in terms of stream order, flow rate, size, and isolation were sampled over a 5-year period, at 3-monthly intervals. All fish and prawns were caught from the stretch, the animals were identified to species and weighed (wet weight to the nearest 0.1 g) using a portable electronic balance, and then returned, unharmed, to the site at which they were captured. Although the sampling included several prawn species, the non-indigenous invasive prawn species *M. rosenbergii* (Mohammed et al. 2011) was not recorded at any of the sites. On the last day of sampling ten female guppies were collected per site, euthanized with an overdose of benzocaine, measured their standard length to the nearest 0.01 mm using digital callipers and placed them in 4% buffered paraformaldehyde. We chose females because the brains of the much smaller males are often folded into bony protuberances of the brain cavity in a way that makes complete extraction impossible (personal observation; Burns and Rodd 2008). Hence, all following results are applicable to females and whether the same patterns apply to males is currently unknown.

### Predation pressure estimation

**Fig. 1:**
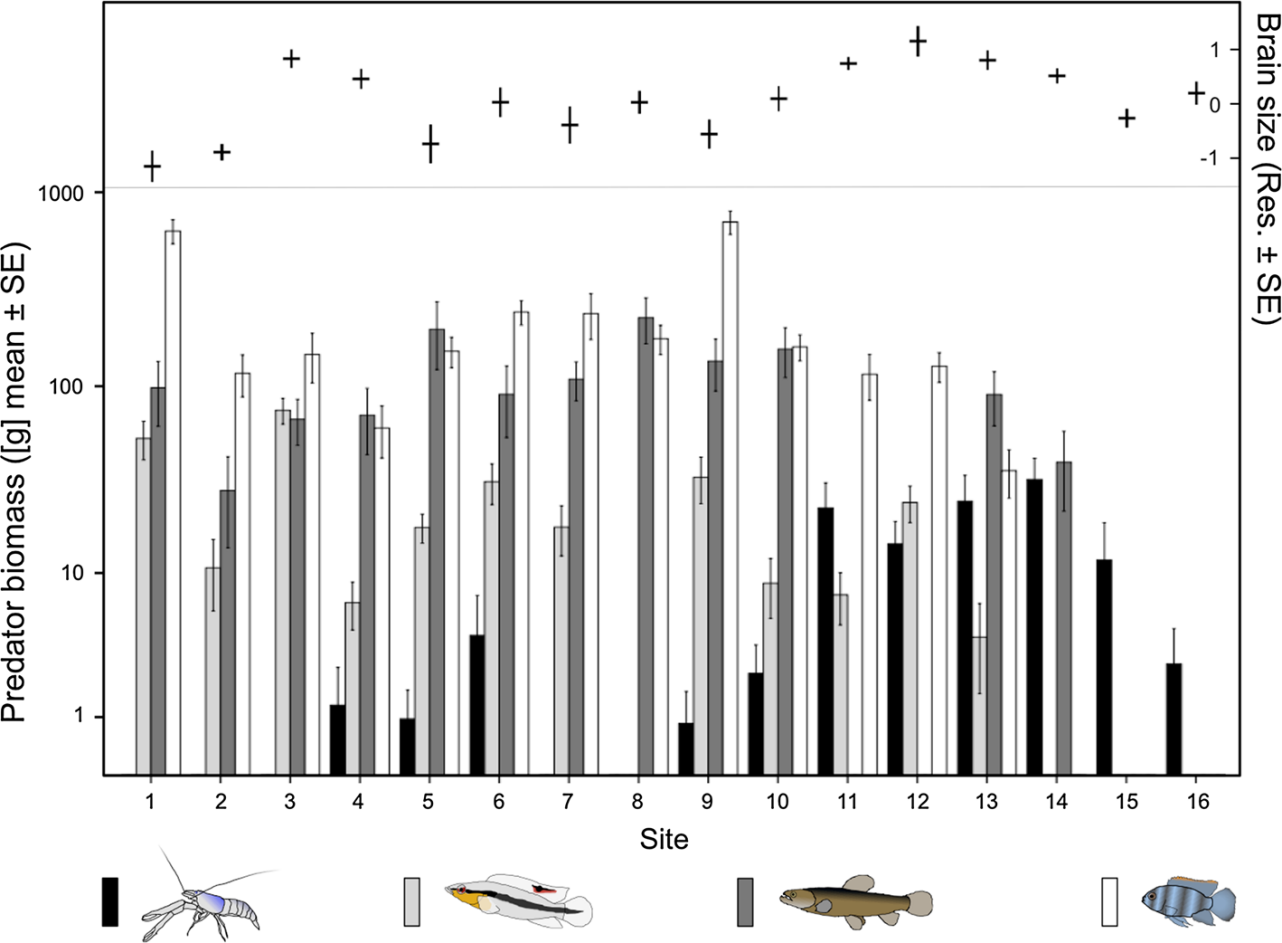
Mean biomass of guppy predators and guppy brain sizes for 16 study sites. The *bars* show the means of 20 censuses over 5 years for pike cichlid (*light grey bars*), wolf fish (*dark grey bars*), freshwater prawn (*black bars*) and blue acara cichlid (*white bars*) on a log10 scale. The *error bars* in the upper part show relative brain sizes (the residuals of a regression of brain mass controlled for body size)

The major predators of guppies in Trinidad in general, and in the chosen populations in particular, are the pike cichlid (*Crenicichla frenata*), the blue acara cichlid (*Andinoacara pulcher*), the wolf fish (*Hoplias malabaricus*), and several morphologically similar large species of freshwater prawns (*Macrobrachium* spp.; Botham et al. 2006; Deacon et al. 2015; Endler 1980; Rodd and Reznick 1991; Seghers 1974). We found several other fish species at those sites, but they are not believed to consume adult guppies (personal communication Rajindra Mahabir). The four major predators comprised 33.4% of the total non-guppy biomass at the sites. These predators differ greatly in hunting strategy, and should therefore pose highly different cognitive demands on their prey. Pike and acara cichlids are medium-sized (10–15 cm), diurnal predators while wolf fish can attain a larger size (up to 50 cm) and usually hunt during dusk, night and dawn (Seghers 1973). Acaras are considered to pose the lowest threat of the four species (Botham et al. 2006). The approx. 9 cm large prawns (carapace and abdomen; Chace 1969) are omnivorous and their role as guppy predators is well established (e.g. Rodd and Reznick 1991); pike cichlids and wolf fish are strictly carnivorous. Wolf fish and prawns hunt using a sit-and-wait, ambush strategy, while the two cichlid species show a more active pursuit strategy (Botham et al. 2006; Seghers 1973). We used the mean biomass of each predatory species per site, computed from 20 censuses, as a proxy for predation pressure. The logic behind this is that a higher biomass of predators needs more food to support its existence (Endler 1978). As the sites are of similar size and topography (Deacon et al. 2015), predator biomass should determine predator pressure. We used only animals >1 g in those calculations, as smaller individuals are unlikely to consume adult guppies. Repeatability was highly significant over the 5-year period (Lessells and Boag 1987): Acara cichlid: r = 0.65, prawns: r = 0.17, pike cichlid: r = 0.43, wolf fish: r = 0.20 (all *p* < 0.001; Fig. 1).

Juvenile guppies are small enough to be consumed by all co-occurring fish species (Seghers 1974, Deacon et al. 2011). A larger diversity of predators should pose a greater cognitive challenge, as more evading strategies need to be mastered. This may be reflected in corresponding brain anatomy differences. To investigate whether the predator diversity indeed impacts brain anatomy we therefore used the mean number of predator species (all co-occurring fish species) per site as indicator of general predator pressure. Repeatability (Lessells and Boag 1987) for this measure was also highly significant (r = 0.68, *p* < 0.001).

### Brain measurements

All dissections, digital image analyses and measurements were performed blind with regards to treatment by one person (AK). We removed the brain from the skull and weighed it to the nearest 0.001 mg. To quantify brain region volumes, digital images of the dorsal, ventral, left and right side of the brain were taken through a dissection microscope (Leica MZFLIII), using a digital camera (Leica DFC 490). For each image, the brain was placed to ensure that it was symmetrically positioned such that one hemisphere did not appear larger than the other based on perspective. For paired regions, both sides were measured and the volumes added to give total region volume. Following Pollen et al. (2007) the widths *W* of six key regions (olfactory bulb, telencephalon, optic tectum, cerebellum, hypothalamus and dorsal medulla) were determined from dorsal and ventral views, whereas lengths *L* and heights *H* were taken from lateral views. The width *W* was defined as the maximal extension of a given region perpendicular to the anatomical midline. The length *L* of a region was defined as the maximal extension of a structure in parallel to the estimated projection of the brain, the height *H* as the maximal extension of the structure perpendicular to the estimated projection of the brain. The volume of the brain regions *V* was determined according to an ellipsoid model (van Staaden et al. 1995).

$$V = \left( {L*W*H} \right)\frac{\pi }{6}$$

This method was recently shown to provide comparable data to more advanced methods such as CT-scanning (White and Brown 2015).

To determine repeatability (Lessells and Boag 1987) the volume of all regions in 10 randomly chosen specimens were measured twice. Repeatability for structures was very high (r = 0.87–0.93, all *p* < 0.001).

### Statistical analysis

We used linear mixed models (LMM) to investigate the effect of predator pressure on the size of the brain and six brain regions. We log-transformed body size ([mm], standard length—measured from the tip of the snout to the end of the caudal peduncle), brain weights [mg] and brain region volumes [mm^3^] to account for potential allometry effects, and centred and standardized all variables (Schielzeth 2010). For brain size we used the mass of the brain as dependent variable, body size as covariate, the mean biomass for each of the four predators as continuous fixed effects and the 16 different sampling sites as random effect. The full model included all 2-way interactions. Instead of stepwise model reduction, which is often prone to subjective bias, we used the program “glmulti” (Calcagno 2013) in R to find the best model fit based on lowest AIC values. For the six brain regions we used an analogous approach in six separate models with the region of interest as dependent variables and the mass of the brain as covariate (Gonda et al. 2009; Kotrschal et al. 2012a, 2014b). To test for the effect of predator diversity on guppy brain anatomy we used analogous LMMs but instead of predator biomass we used the mean number of fish species per site as covariate. To facilitate readability of the following text, we simplified the wording where appropriate: (1) Brain mass is corrected for body size and brain regions are corrected for brain mass (see above), therefore all results represent *relative* brain size and *relative* brain region sizes. We will omit the ‘relative’ from hereon. (2) For the effects of predators, the biomass of the respective predators is used in all analyses, but we omit ‘biomass’ in appropriate cases from hereon.

## Results

**Fig. 2:**
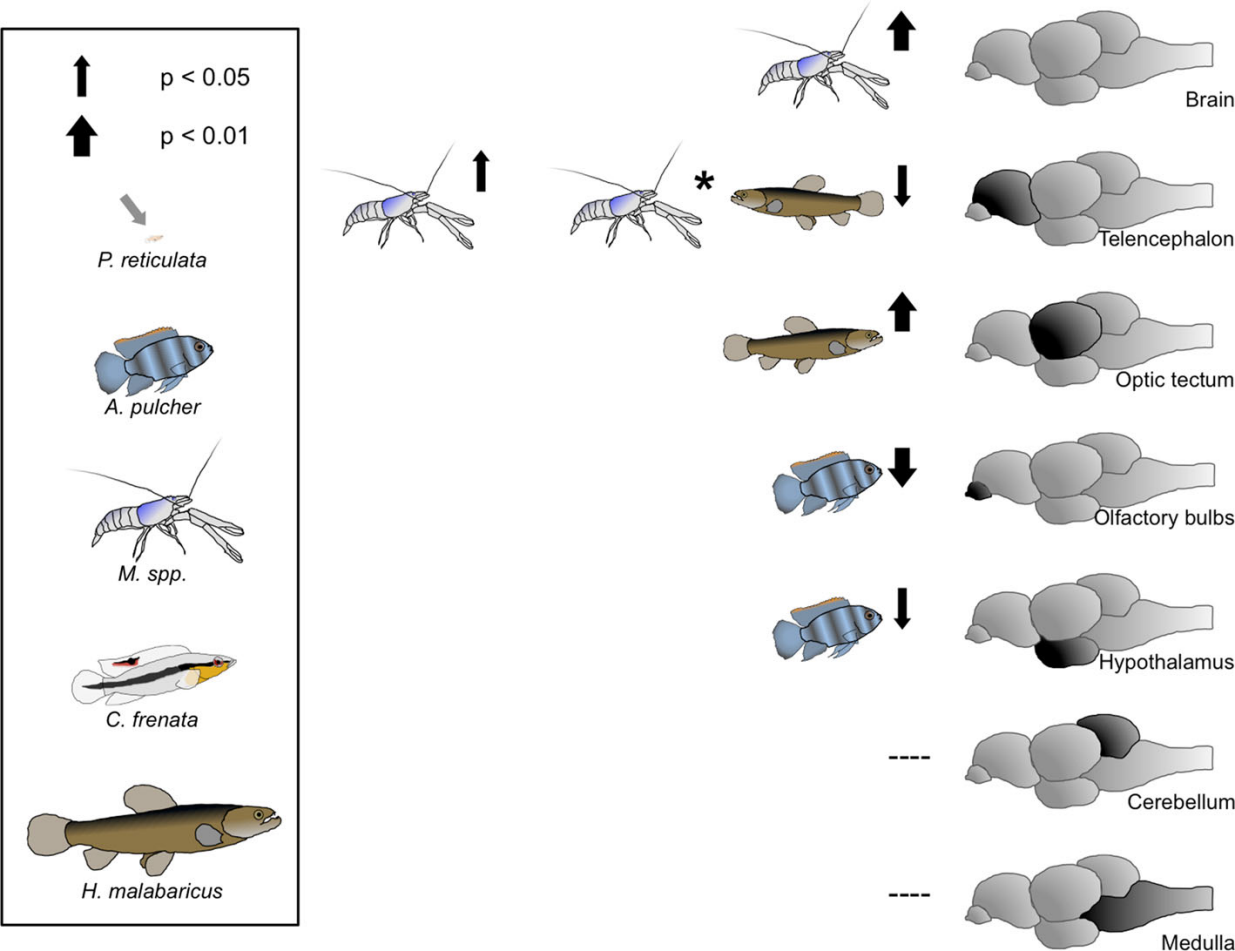
Schematic representation of the impact of the abundance of the four major guppy predators on female guppy brain anatomy. The four predators, blue acara cichlid (*Andinoacara pulcher*), pike cichlid (*Crenicichla frenata*), wolf fish (*Hoplias malabaricus*), and freshwater prawn (*Macrobrachium* spp.) on the *left* are to scale with an adult guppy female. Orientation of the *arrows* indicates positive/negative associations; *thickness* indicates the strength of the association

**Fig. 3:**
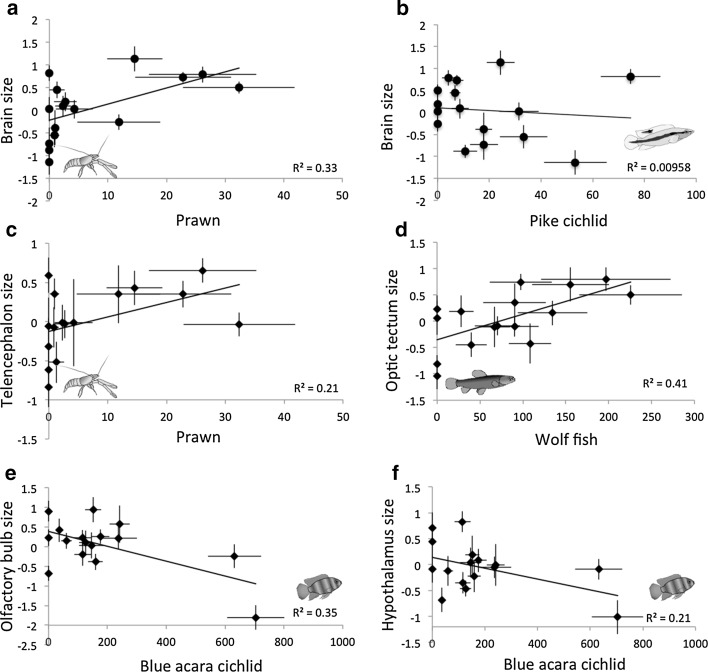
The relationship between the biomass of predators in 16 sites and the relative brain and brain region sizes of female guppies from those sites. Whole brain size (**a**) and telencephalon size (**c**) are positively associated with prawn biomass, optic tectum size (**d**) is positively associated with wolf fish biomass, while olfactory bulbs (**e**) and hypothalamus size (**f**) are negatively associated with blue acara cichlid biomass. Whole brain size and pike cichlid biomass (**b**) are not associated. The y-axes show the mean relative brain anatomy measures (residuals ± S.E.; brain size corrected for body size, brain region sizes corrected for brain size), the x-axes show the mean of 20 samplings per site (±S.E.)

Of the 16 sites, acara cichlids were present at 13, pike cichlids and wolf fish at 12, and prawns at 11; six sites harboured all four predators simultaneously, while at two sites only prawn predators were found. Overall, besides guppies we found on average between 3.2 and 10.2 fish species per site. The mean predator biomass and the brain size of guppies varied considerably among sites (Fig. 1). We found that brain size was significantly positively correlated with prawn biomass across 16 populations of guppies. This was true for a model with only prawns as factor (LMM_brain_: body size: DF = 160.2, t = 45.4, *p* < 0.001; prawns: DF = 160.5, t = 2.81, *p* = 0.0125, AIC = −735.6), but glmulti revealed that brain size was best explained if the effects of blue acara and pike cichlid were also accounted for. In addition to the significant positive effect of prawns on guppy brain size (*p* = 0.0035), this full model (AIC-740) revealed that brain size tends to increase with pike cichlids (*p* = 0.0955) and that blue acara cichlids tend to dampen the prawn effect (acara * pike cichlid, *p* = 0.0697, Table 1, Figs. 2, 3a). Predator diversity was not associated with relative brain size (*p* = 0.573, Table 2).

**Table 1:** The effect of predator biomass on whole brain and brain region size of guppy females from 16 populations

	Estimate	SE	df	t value	*p*
**Whole Brain**					
Body size	0.127692	0.002638	119.33	48.41	<0.001
Prawn	0.013547	0.004008	16.85	3.38	0.0035
Blue acara cichlid	−0.00469	0.006372	14.71	−0.74	0.4731
Pike cichlid	0.007495	0.004220	15.35	1.78	0.0955
Acara * pike	−0.009879	0.005050	14.71	−1.96	0.0697
**Telencephalon**					
Brain size	0.130635	0.002895	179	45.13	<0.001
Prawn	0.006873	0.003314	179	2.07	0.0395
Wolf fish	0.004017	0.003070	179	1.31	0.1925
Prawn * wolf fish	−0.010813	0.004741	179	−2.28	0.0237
**Optic tectum**					
Brain size	0.119568	0.002194	94.78	54.49	<0.001
Wolf fish	0.007805	0.002571	16.16	3.04	0.0078
**Olfactory bulbs**					
Brain size	0.120231	0.007028	157.78	17.11	<0.001
Blue acara cichlid	−0.036563	0.012056	15.52	−0.033	0.0081
**Hypothalamus**					
Brain size	0.136334	0.003908	95.02	34.88	<0.001
Prawn	0.008325	0.004697	19.07	1.77	0.0923
Blue acara cichlid	−0.010185	0.004532	13.80	−2.247	0.0415
**Cerebellum**					
Brain size	0.1569	0.004746	111.00	33.07	<0.001
**Medulla oblongata**					
Brain size	0.1534	0.004444	143.20	34.52	<0.001

Shown are the results of the best general linear mixed effect models according to lowest AIC

**Table 2:** The effect of predator diversity on whole brain and brain region size of guppy females from 16 populations

	Estimate	SE	DF	t value	*p*
**Whole brain**					
Body size	1.6016	0.0364	173.2	44.03	<0.001
Predator diversity	−0.0016	0.0029	14.6	0.57	0.573
**Telencephalon**					
Brain size	1.0106	0.0219	122.5	45.98	<0.001
Predator diversity	0.0009	0.0019	13.6	0.64	0.635
**Optic tectum**					
Brain size	0.9071	0.0177	116.8	51.18	<0.001
Predator diversity	0.0032	0.0014	16.2	2.26	0.038
**Olfactory bulbs**					
Brain size	0.9136	0.563	175.0	16.23	<0.001
Predator diversity	−0.0013	0.0074	15.0	−0.17	0.865
**Hypothalamus**					
Brain size	1.0464	0.0324	132.3	32.31	<0.001
Predator diversity	−0.0025	0.0028	15.5	−0.89	0.388
**Cerebellum**					
Brain size	1.1749	0.0376	128.7	31.2	<0.001
Predator diversity	−0.0025	0.0032	14.7	−0.78	0.448
**Medulla oblongata**					
Brain size	1.1552	0.0349	156.3	33.11	<0.001
Predator diversity	−0.0006	0.0035	14.7	−0.17	0.871

Shown are the results of linear mixed effect models

For the brain regions, we found that telencephalon size was positively correlated with prawn biomass (*p* = 0.0395, Table 1, Figs. 2, 3c). Wolf fish did not influence telencephalon size as such (*p* = 0.1925, Table 1), but they dampened the relationship between telencephalon size and prawns, as indicated by a significant interaction (prawns * wolf fish, *p* = 0.0237, Table 1, Fig. 2). Optic tectum size was strongly positively correlated with wolf fish biomass only (*p* = 0.0078, Table 1, Figs. 2, 3d), while the size of the olfactory bulbs correlated negatively with blue acara cichlid biomass (*p* = 0.0081). The size of the hypothalamus was negatively correlated with blue acara biomass (*p* = 0.0415, Table 1, Figs. 2, 3d), but also showed a non-significant trend towards a positive correlation with prawn biomass (*p* = 0.0923). Predator diversity was positively associated to optic tectum size only (*p* = 0.038, Table 2).

## Discussion

Female guppies from areas with higher prawn biomass had a larger brain and larger telencephalon. Individuals from areas with higher wolf fish biomass had a larger optic tectum, and those from areas with higher acara cichlid biomass had smaller olfactory bulbs and a smaller hypothalamus. Animals in areas with greater fish species number also showed larger optic lobes. Because we chose sites that are similar in important ecological and abiotic factors, we suggest that the varied cognitive challenges posed by the different predators exert divergent selection pressures that underlie the observed differences in brain anatomy. Below we discuss how our results support the hypothesis that predation ecology is an important selective force in the evolution of brain anatomy and the general implications of this finding.

In line with our predictions, one of our most salient findings was that specific aspects of predation pressure and larger brains are positively associated in the wild. Combined with laboratory-based findings showing that a larger brain is advantageous in evading predation (Kotrschal et al. 2015a; van der Bijl et al. 2015), this corroborates the hypothesis that by consuming smaller-brained individuals some predators can inadvertently select for large brain size. Based on this we hypothesize that in settings where prey is evolving larger brains in response to predation, this should exert selective pressure on the predators. Evidence for such a cognitive arms race between prey and predator comes from a large-scale comparison of 623 prey-predator species pairs in fishes (Kondoh 2010), which showed that larger-brained predators tend to target larger-brained prey. Consequently, a larger brain size may lead to greater behavioural flexibility (Lefebvre et al. 2004; Sol et al. 2005) and allow predators to feed on additional prey species. This is exactly what has recently been shown in a Canadian lake; that species tend to evolve larger brains with increasing relative trophic position in the food web (Edmunds et al. 2016). However, the costs of developing a large brain and the general complexity of food webs likely place limits on such relationships. The costs of large brains include a decreased reproductive output (Kotrschal et al. 2013a) and longer juvenile period (Hawkes et al. 1998; Kotrschal et al. 2015b), both of which are important factors when it comes to the impact of predation (Sogard 1997). As predators usually prey on several different species, it is possible that they simply stop feeding on one species if it evolves large-enough brains to “outsmart” them. Additionally, and non-mutually exclusively, prey species may evolve other forms of anti-predator strategies such as group living (Pulliam and Caraco 1984), which has been suggested to be associated with increased brain size (Dunbar 1998; but see van der Bijl and Kolm 2016 for discussion of this topic). In the case of the guppy it is apparent that it is primarily prawn predation that impacts brain mass, while the fish predators seem to play a less prominent role. We found a very similar result for the telencephalon, which likely drives this whole brain size effect (see below). We found only a non-significant trend linking pike cichlid biomass and guppy brain size. This is somewhat surprising, as pike cichlids are generally regarded as the most important guppy predator and the model predator species when it comes to experiments on guppy ecology (Endler 1980; Kotrschal et al. 2015a). Our findings indicate that prawns deserve greater consideration in such studies. We found no support for the idea that a predator species rich community would select for larger brains.

For brain regions we found two major effects of predation pressure, which are both in line with our predictions: the positive associations between telencephalon size and prawn biomass, and between optic tectum size and wolf fish biomass. The telencephalon in teleost fish, like that of mammals, is, important in learning and memory (reviewed in Overmier and Hollis 1983). We therefore suggest that the challenges of prawn predation can be met by increasing those learning aspects of cognition. More specifically, in contrast to all the fish species that prey on guppies, prawns cannot consume their prey in one go; their mouth apparatus allows only for piecewise consumption. By observing this process, bystanders have the opportunity to learn about the dangers of prawn predation via associative learning (Brosnan et al. 2003). This means that an increased learning ability should confer a survival benefit, which may underlie the effect of prawn biomass on telencephalon size as well as on overall brain size as suggested above. Intriguingly, we also found an interaction between prawn and wolf fish biomass that negatively affected telencephalon size. Wolf fish presence hence dampens and/or removes the positive effect of prawn predation on telencephalon size. Potentially because a higher wolf fish density decreases the cognitive advantage that a larger telencephalon confers when guppies learn about the dangers of prawn predation; observing the consumption of conspecifics may be too dangerous in habitats with high wolf fish presence. This however needs to be investigated further, as a study on anti-predator behaviours of guppies presented with wolf fish as well as pike and acara cichlids found no qualitative differences in guppy responses towards wolf fish versus cichlids (Botham et al. 2006).

The optic tectum receives and integrates visual information and even though its integrity is not necessary for many basic aspects of visual perception, such as phototaxis (Ullén et al. 1997), optomotor response (Roeser and Baier 2003), or detection of stationary barriers (Ingle 1973), its crucial role in motion detection is vital for predator detection and fleeing response (reviewed in Roeser and Baier 2003). We therefore attribute the strong positive association between optic tectum size and wolf fish biomass to the ambush hunting behaviour of wolf fish. While better visual acuity is unlikely to confer a benefit when being chased by a predator (e.g. a cichlid), it is likely that a larger optic tectum facilitates early detection of the sudden movements of a predator that sits in ambush. This should lead to a faster initiation of a C-start escape and more efficient evasion. Future experiments should test whether a relatively larger optic tectum indeed confers survival benefits when faced with such predators. The fact that we found an association between predator diversity and optic tectum size further highlights its potential role in predator evasion. Guppies adopt individual evasion strategies for some predators such as increased schooling or predator inspection (Botham et al. 2006). Potentially, if the diversity of predators gets too large, species-specific strategies may no longer be cognitively beneficial and animals resort to a general “flee early” evasion strategy. This may explain larger optic tectum with greater predator diversity.

Blue acara cichlids are considered the least serious predators to guppies and it is intriguing that their biomass seems to be negatively associated with both olfactory bulb and hypothalamus size. Those results seem to be driven by a single site where blue acara cichlids are very common and both the olfactory bulbs and the hypothalamus are exceptionally small. Indeed, if this site is removed from the data set, olfactory bulb and hypothalamus size are not significantly related to acara cichlid biomass (both *p* > 0.1). It is therefore too early to speculate whether there is an overall relationship between those brain regions and acara biomass, and on the potential mechanism that may underlie such a relationship.

As in all correlative studies, we cannot infer causality from our results as some unknown third factor may underlie both variation in predator community and variation in brain anatomy. However, at least two aspects argue for causality in our case. Firstly, experiments have shown that guppy females artificially selected for large and small brain size differ markedly in both antipredator behaviour (van der Bijl et al. 2015) and survival under predation (Kotrschal et al. 2015a). Secondly, the sites in our study were carefully chosen to be as similar as possible to each other and consequently the abiotic factors that were assessed were highly comparable (Deacon et al. 2015).

If we accept that population differences in female guppy brain anatomy are likely to be causally linked to differences in predation ecology, what is the underlying mechanism? Is it the result of predator-driven local adaptations (i.e. evolution), or of experience-dependent plasticity, or a combination of the two? The literature provides examples in support of both scenarios. For instance, local adaptation for large hippocampus size in chickadees (*Parus rufescens*) is thought to be the basis for enhanced food caching (Croston et al. 2015), while hippocampus size in London taxi drivers increases with job experience (Maguire et al. 2006; Woollett and Maguire 2011). And nine-spine sticklebacks (*Gasterosteus aculeatus*) show plastic responses in brain region size in relation to perceived predation pressure (Gonda et al. 2012). Although guppy brains show phenotypic plasticity (Burns et al. 2009b; Kotrschal et al. 2012a), Trinidadian guppies are a textbook example of local adaptations in multiple traits (Bassar et al. 2010). If reared in a common garden setting, guppies from different populations often keep their differences in body morphology (Burns et al. 2009a), behaviour (O’Steen et al. 2002; Seghers 1974) and most importantly brain anatomy (Burns and Rodd 2008). Further common garden experiments are needed to determine the degree to which local adaptation and phenotypic plasticity underlie the observed differences in brain anatomy. Meanwhile, it is parsimonious to attribute a considerable proportion of the site-specific brain anatomy differences to the evolutionary history of the populations.

In conclusion, we show that predation is associated with brain anatomy variation in wild populations. Our study thus provides support for the longstanding hypothesis that in challenging situations, natural selection favours individuals with larger brains. We suggest that a change in brain anatomy may facilitate anti-predator strategies via changes in specific aspects of cognitive ability and our study identifies predation pressure as a key selective pressure in brain evolution in natural populations.
